# Dry season temperature and rainy season precipitation significantly affect the spatio-temporal pattern of rubber plantation phenology in Yunnan province

**DOI:** 10.3389/fpls.2023.1283315

**Published:** 2023-12-14

**Authors:** Hongyan Lai, Bangqian Chen, Xiong Yin, Guizhen Wang, Xincheng Wang, Ting Yun, Guoyu Lan, Zhixiang Wu, Chuan Yang, Weili Kou

**Affiliations:** ^1^ College of Forestry, Southwest Forestry University, Kunming, China; ^2^ Hainan Danzhou Agro-ecosystem National Observation and Research Station, State Key Laboratory Incubation Base for Cultivation & Physiology of Tropical Crops, Rubber Research Institute (RRI), Chinese Academy of Tropical Agricultural Sciences (CATAS), Haikou, China; ^3^ Co-Innovation Center for Sustainable Forestry in Southern China, Nanjing Forestry University, Nanjing, China

**Keywords:** global warming, rubber plantations, S-G filter, GEE, phenology change, legacy effect

## Abstract

The ongoing global warming trajectory poses extensive challenges to plant ecosystems, with rubber plantations particularly vulnerable due to their influence on not only the longevity of the growth cycle and rubber yield, but also the complex interplay of carbon, water, and energy exchanges between the forest canopy and atmosphere. However, the response mechanism of phenology in rubber plantations to climate change remains unclear. This study concentrates on sub-optimal environment rubber plantations in Yunnan province, Southwest China. Utilizing the Google Earth Engine (GEE) cloud platform, multi-source remote sensing images were synthesized at 8-day intervals with a spatial resolution of 30-meters. The Normalized Difference Vegetation Index (NDVI) time series was reconstructed using the Savitzky-Golay (S-G) filter, coupled with the application of the seasonal amplitude method to extract three crucial phenological indicators, namely the start of the growing season (SOS), the end of the growing season (EOS), and the length of the growing season (LOS). Linear regression method, Pearson correlation coefficient, multiple stepwise regression analysis were used to extract of the phenology trend and find the relationship between SOS, EOS and climate factors. The findings demonstrated that 1) the phenology of rubber plantations has undergone dynamic changes over the past two decades. Specifically, the SOS advanced by 9.4 days per decade (R^2^ = 0.42, *p*< 0.01), whereas the EOS was delayed by 3.8 days per decade (R^2^ = 0.35, *p*< 0.01). Additionally, the LOS was extended by 13.2 days per decade (R^2^ = 0.55, *p*< 0.01); 2) rubber phenology demonstrated a notable sensitivity to temperature fluctuations during the dry season and precipitation patterns during the rainy season. The SOS advanced 2.0 days (r =−0.19, *p*< 0.01) and the EOS advanced 2.8 days (r =−0.35, *p*< 0.01) for every 1°C increase in the cool-dry season. Whereas a 100 mm increase in rainy season precipitation caused the SOS to be delayed by 2.0 days (r = 0.24, *p*< 0.01), a 100 mm increase in hot-dry season precipitation caused the EOS to be advanced by 7.0 days (r =-0.28, *p*< 0.01); 3) rubber phenology displayed a legacy effect of preseason climate variations. Changes in temperature during the fourth preseason month and precipitation during the fourth and eleventh preseason months are predominantly responsible for the variation in SOS. Meanwhile, temperature changes during the second, fourth, and ninth preseason months are primarily responsible for the variation in EOS. The study aims to enhance our understanding of how rubber plantations respond to climate change in sub-optimal environments and provide valuable insights for sustainable rubber production management in the face of changing environmental conditions.

## 1 Introduction

Plant phenology—capturing the recurrent dynamics of flora in response to annual climatic and environmental changes—represents a fundamental biological pattern (Reich, 1995; Körner and Basler, 2010; Li X. et al., 2016). A nuanced understanding of the interplay between plant phenology and evolving climate patterns can shed critical light on the prospective effects of climate change, particularly with regard to vegetation ecosystems structure, functioning, and ecosystem services offerings (Stöckli and Vidale, 2010). With the backdrop of escalating global warming, disparate vegetation types demonstrate remarkably diverse phenological responses, underscoring the complexity of these ecological systems (Yoshifuji et al., 2006; Tan et al., 2011; Gong et al., 2022). Consequently, this dynamic interrelation between varying vegetation phenology and the climate represents a prominent nexus in contemporary ecological and climate change research, calling for increased scholarly attention (Huang et al., 2013; Fitchett et al., 2015; Zhang et al., 2023).

Rubber trees (*Hevea brasiliensis*), as a perennial deciduous species, are prominently known for natural rubber production (Chiarelli et al., 2020). These trees undergo a rapid defoliation and refoliation during the dry season in the Northern Hemisphere, typically achieving full defoliation around February (Rao et al., 1998; Azizan et al., 2021a). Traditional ground-based phenology observations remain essential for various management tasks, including seedling breeding, disease control, latex harvesting, and yield forecasting (Chen et al., 2006; Lin et al., 2016; Li et al., 2019; Yin et al., 2022). Nevertheless, with the rapid advancement of remote sensing technology, remote sensing data, with its advantages of broad coverage, long-term time series, and reduced interference, have become the primary method for rubber tree phenology monitoring (Li et al., 2018). Remote sensing technology has greatly contributed to the mapping of rubber plantations, observing spatial and temporal dynamic changes, and developing climate prediction models (Che et al., 2014; Kou et al., 2015; Razak et al., 2018; Li Y. et al., 2020; Xiao et al., 2020; Xiao et al., 2021). The majority of tropical plants lack long-term phenological field dates and seasonal variation is not readily apparent (Yoshifuji et al., 2006; Tan et al., 2011; Gong et al., 2022). Consequently, our understanding of how tropical vegetation responds to global climate change remains considerably constrained (Abernethy et al., 2018; Qian et al., 2021). The extensive distribution and distinct phenological stages make rubber tree ideal subjects for studying phenology in tropical regions (Guyot et al., 2001; Lin et al., 2022). Acquiring accurate phenology data through remote sensing enhances management’s ability to forecast production activities, providing valuable insights into the response of tropical vegetation to climate change.

Yunnan Province, the principal rubber-producing base in China, is positioned within the sub-optimal cultivation zones for rubber plantations in Southeast Asia, marking it an area of distinct ecological interest (Zhu et al., 2006; Yu et al., 2014; Chen B. et al., 2023). The rubber plantations therein are located within semi-humid tropical climates, characterized by comparatively lower annual temperatures and less abundant precipitation than their humid tropic counterparts (Kou et al., 2015; Liyanage et al., 2019; Zhai et al., 2019; Zhai and Xu, 2022) These conditions substantially deviate from traditional rubber cultivation climates, thereby imposing stressors such as low temperatures, dry periods, and high altitudes on rubber tree growth (Liyanage et al., 2016; Tian et al., 2018). Recent studies suggest that temperature and precipitation critically shape phenological indicators and latex yield (Lin et al., 2018; Liyanage et al., 2019; Zhai et al., 2019). For instance, in the Xishuangbanna, lower temperatures in December expedite the start of the growing season (SOS), whereas higher precipitation in February delays the end of the growing season (EOS) (Zhai et al., 2019; Zhai and Xu, 2022). Moreover, increased sunlight hours during the rainy seasons (May-October) and cold dry seasons (November-February) promotes the advancement of both SOS and EOS (Liyanage et al., 2019). The advent of cold stress, notably temperatures below 10°C, predominantly triggers rubber tree defoliation (Lin et al., 2018; Zhai et al., 2023).

While there is a rubber of phenological research concerning the Xishuangbanna region, there is a conspicuous dearth of studies exploring the inter-annual variation trends of rubber tree phenology within Yunnan (Liu W. et al., 2013; Tan et al., 2014; Xiao et al., 2021; Chen G. et al., 2023). Furthermore, the potential influence of global climate change on rubber tree phenology remains unclear (Liyanage et al., 2019; Zhai et al., 2019; Lin et al., 2022). Therefore, acquiring refined phenological data is imperative to devise comprehensive management strategies for Yunnan’s rubber plantations, as well as predicting potential impacts of climate change on rubber tree phenology. This study set out to execute an extensive examination of the inter-annual phenological variation in Yunnan’s rubber plantations and to quantify its susceptibility to climatic variables. This endeavor employed a robust set of multi-source remote sensing data and meteorological records for the 2001-2020 period. The specific objectives of this research include: 1) evaluating the feasibility and precision of extracting rubber phenological indicators from remote sensing data; 2) analyzing inter-annual variation trend of rubber phenology since 21^th^ century; and 3) revealing the potential influences of climate change on rubber phenology. The insights derived from this investigation have the potential to substantially contribute to the optimization of rubber plantation management practices and the broader progression of the rubber industry.

## 2 Materials and methods

### 2.1 Study area

**Figure 1:**
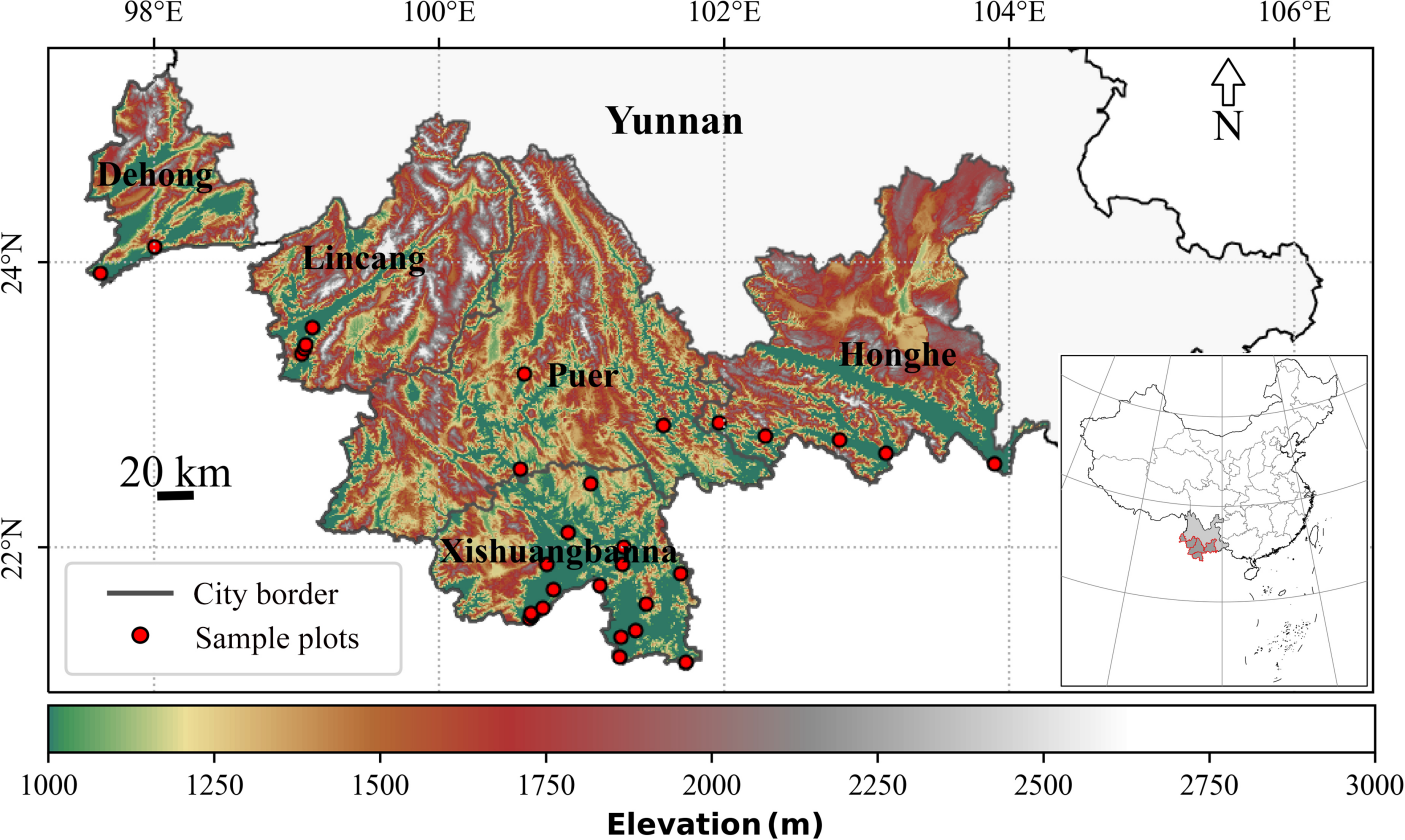
Topography and spatial distribution of sample plots in the study area.

The study region encompasses the rubber plantation area of Yunnan, spanning latitudes 21°10′N to 25°20′N and longitudes 97°30′E to 104°50′E. Specifically, this includes the prefectures of Xishuangbanna, Puer, Lincang, Honghe, and Dehong, as illustrated in **Figure 1**. The region’s topography is characterized by significant variations in elevation, ranging from 200 to 1200 meters above sea level. The prevalent mean annual temperature falls between 19.4 and 27.6°C, whereas annual precipitation oscillates between 1200 to 1900 millimeters (Zhai et al., 2019; Zhai et al., 2023). The regional climate demonstrates distinct seasonality, encompassing a rainy season (May to October), cool-dry season (November to February), hot-dry season (March to April) each year (Lin et al., 2018; Lin et al., 2022; Zhai and Xu, 2022). The SOS and the EOS of rubber plantations occur during the dry season (November to April), a period typically marked by diminished precipitation and lower temperatures (Cao et al., 2006; Liu et al., 2007; Yu et al., 2014; Lin et al., 2018). Yunann rubber plantations in semi-humid tropical climates face adverse conditions that are different from the ideal conditions for rubber cultivation, which are typically found in humid tropical regions.

### 2.2 Data and processing

#### 2.2.1 Field data

A selection of thirty-two representative sample points was made at random, informed by high-resolution satellite imagery from Google Earth. Considerations were given to essential characteristics, such as the stand age (> 4) and altitude of rubber plantations (Lin et al., 2022), ensuring an accurate representation of the plantation landscape. To validate the satellite-derived phenological indicators, we employed field-based data on SOS (refoliation, where leaves transition from copper-brown to light green) and EOS (defoliation, where leaves shift from light yellow to almost completely yellow). These data were collected at the Jinghong Farm (21°48′N, 100°46′E) from 2003 to 2011 (Zhai et al., 2019; Zhai et al., 2020; Zhai et al., 2021) and from the Dongfeng Farm in Jinghong City (21°31′N, 100°39′E) from 2003 to 2020. This rigorous validation process, which involves contrasting satellite-derived phenological indicators with field-based data, reinforces the accuracy and reliability of remote sensing monitoring for phenological evaluations in Yunnan’s rubber plantations.

#### 2.2.2 Satellite imagery

Landsat 5/7/8 (Collection 2 Level 2 Tier 1, 30-m resolution), Sentinel-2 (Level-2A, 20-m resolution), and the moderate-resolution imaging spectroradiometer (MODIS, MOD09GQ and MYD09GQ, v6.1, 250-m resolution) surface reflectance images from 2001 to 2020 were used to extract phenological indicators. These images underwent temporal, spatial, and spectral preprocessing on the Google Earth Engine (GEE) cloud platform. Clouds and shadows in Landsat images were masked using the QA_PIXEL band, while Sentinel-2 images were masked with a scene-tuned cloud probability layer created by the Sentinel-2 Cloud-Detector library (employing LightGBM) (Chen et al., 2021). Clouds and shadows in MODIS images were masked by the associated quality control band (QC_250m) and 0.01 ≤ sur_refl_b01 ≤ 0.25 through simple testing and rounding. The NDVI was calculated according to equation (1) (Tucker, 1979).


(1) $$NDVI=\frac{\rho_{NIR}-\rho_{red}}{\rho_{NIR}+\rho_{red}}$$

where *ρ_red_* and *ρ_NIR_* are red and near-infrared (NIR) band of Landsat, Sentinel-2, and MODIS images, respectively.

#### 2.2.3 Climate data

Precipitation data was sourced from Climate Hazards Group InfraRed Precipitation with Station data (CHIRPS), a quasi-global view of precipitation patterns with a spatial resolution of 0.05 rad and a temporal resolution of 1 day (Shen et al., 2020). Temperature data was obtained from the MOD11A1 and MYD11A1 daily Land Surface Temperature (LST) product, which offer a high spatial resolution of 1 km (Metz et al., 2017). Through the utilization of a geemap-based script, annual and monthly precipitation and temperature values were meticulously calculated and extracted for sample points from 2001 to 2020 (Peterson et al., 2013; Noi et al., 2016). Monthly preseason temperature and precipitation were employed to investigate their relationships with rubber phenology and determine the optimum climate preseason for rubber phenology (Cho et al., 2017; Ren et al., 2017; Zhai et al., 2019).

### 2.3 Methodology

#### 2.3.1 Method overview

**Figure 2:**
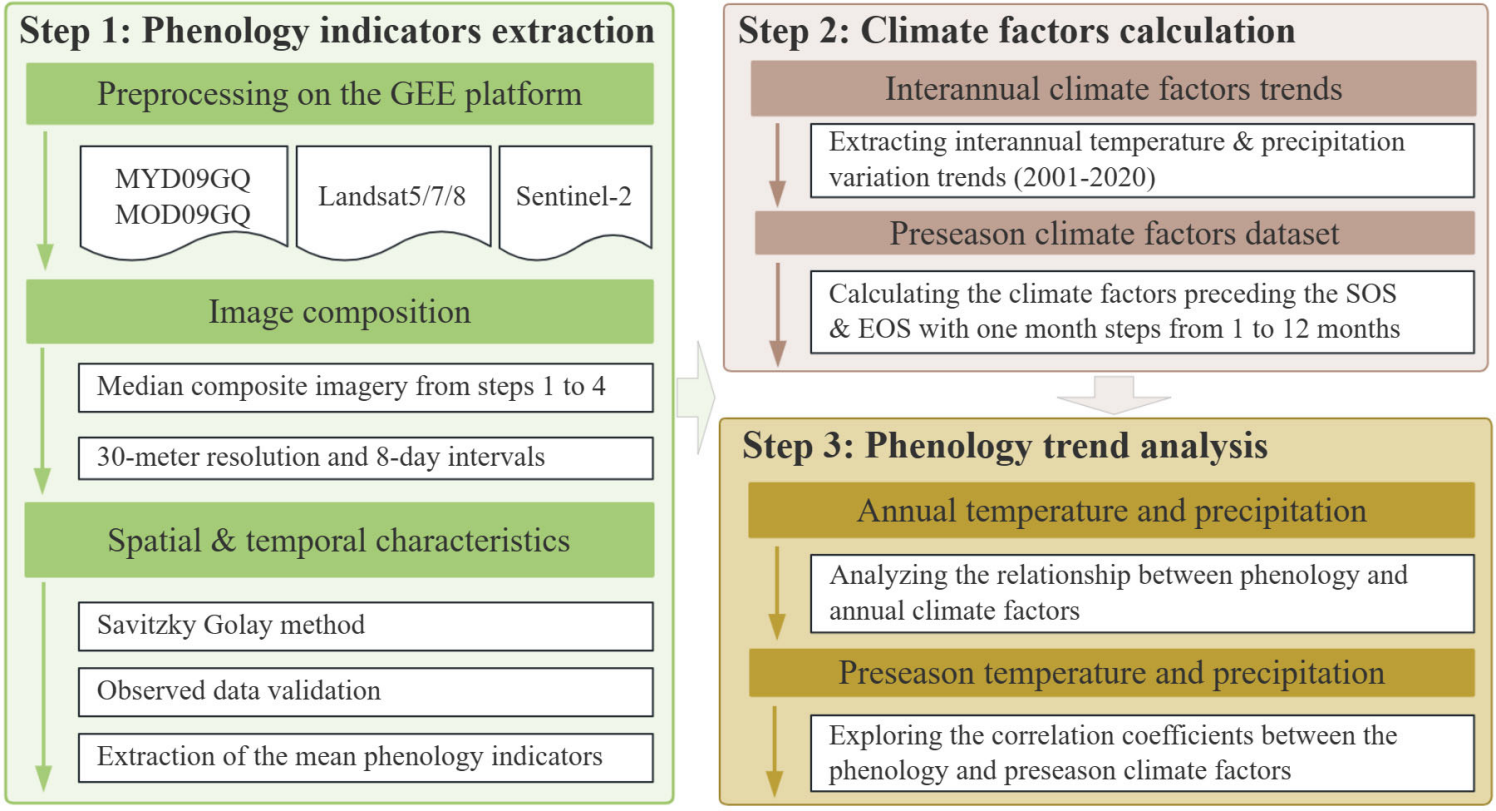
Flowchart of processes: (step 1) phenology, (step 2) climate, and (step 3) phenology trend analysis.

The processes are divided into three main sections: 1) phenological indicators extraction; 2) climate factors calculation; and 3) phenology trend analysis. Each process is explained in detail in the following subsections (**Figure 2**).

#### 2.3.2 Construction of NDVI time series

Annual NDVI time series at 30-m resolution and 8-day intervals were produced using Landsat, Sentinel-2, and MODIS imagery on the GEE platform. The annual image composition commenced on January 1, producing a scene every eight days. The final scene was supplemented with images from the following year to ensure 46 scenes annually. Landsat and Sentinel-2 (LS2) median composite imagery was primarily employed instead of 250-m MODIS imagery, as rubber plantations are typically situated in highly fragmented landscapes (Azizan et al., 2021a; Yang et al., 2021; Chen G. et al., 2023). As the temporal range of Landsat imagery far exceeds that of Sentinel-2, the resolution of the composite imagery was harmonized to match the 30-m Landsat resolution. The composition algorithm is described as follows: 1) mean NDVI of LS2 images acquired in the specific 8-day window of the current year, 2) mean NDVI of LS2 images acquired in the previous and subsequent year of the same 8-day window when step 1 failed (no cloud-free image), 3) mean NDVI of MODIS (upscale to 30-m by resampling) images acquired in the specific 8-day window of the current year when step 2 failed, and 4) mean NDVI of MODIS images acquired in the previous and subsequent year of the same 8-day window when step 3 failed. Excluding the critical defoliation stage of rubber plantations (Day of Year, DOY< 15 or DOY > 75), a rapid drop (here -0.12 based on simple tests) in NDVI in the adjacent 8-day window was considered an outlier and replaced with the maximum NDVI value from steps 1 to 4. The new compositional algorithm is suitable for the whole study area for each year (**Figure S1**). All composite images were downloaded to a local computer for TIMESAT analysis.

#### 2.3.3 Determination of SOS and EOS thresholds

Specifically, the SOS was defined as the time when the terminal buds of rubber trees underwent a noticeable transition from coppery brown to light green, signifying a significant increase in NDVI. Conversely, the EOS represented the time when the light-yellow leaves of the rubber trees transformed to an almost complete yellow color, marking a substantial decrease in NDVI. The LOS was defined as the duration between SOS and EOS. The S-G filter was employed to mitigate noise and remove outliers from the NDVI time series (Glade et al., 2016). Utilizing the seasonal amplitude method for extracting phenological indicators allowed for circumventing some limitations associated with the absolute threshold method (Jönsson and Eklundh, 2002).

#### 2.3.4 Statistics analysis

To unveil the spatial variation of rubber phenology, we employed the mean value method. This approach was used to calculate the multi-year mean values of rubber phenology indicators. Furthermore, we evaluated the trends of rubber phenology indicators, temperature, and precipitation by calculating the slopes (k) and coefficients of determination (R^2^) (Eq. 2&3). In order to quantitatively evaluate the climate response to preseason climate change and to probe the potential temporal legacy effect on rubber phenology, we computed the partial correlation coefficients of month-by-month mean temperature and total monthly precipitation corresponding to the dates of SOS and EOS, from the preceding first to twelfth month, using SPSS software (Azizan et al., 2021a; Azizan et al., 2022) (Eq. 4). Additionally, we conducted multiple stepwise regression analysis. During model construction, we considered the contribution of each factor to the model and quantified its significance using R^2^ and relative contribution. This approach allowed us to quantitatively assess the degree to which each climatic factor significantly influences rubber phenology. Python package such as Matplotlib (https://matplotlib.org) and Seaborn (https://seaborn.pydata.org) were used to plot various figures (Waskom, 2021).


(2) $$K=\frac{N\sum {\;}_{i=1}^{N}(iG_{i})-(\sum {\;}_{i=1}^{N}i)(\sum {\;}_{i=1}^{N}G_{i})}{\text{N}\sum {\;}_{i=1}^{N}i^{2}-{\left(\sum {\;}_{i=1}^{N}i\right)}^{2}}$$

(3) $$R^{2}=\frac{\sum {\;}_{i=1}^{n}{\left(y_{i}-{\hat{y}}_{i}\right)}^{2}}{\sum {\;}_{i=1}^{n}{\left(y_{i}-{\bar{y}}_{i}\right)}^{2}}$$

(4) $$r_{xy[z]}=\frac{r_{xy}-r_{xz}\times r_{zy}}{\sqrt{\left(1-r_{xz}^{2}\right)}\sqrt{\left(1-r_{zy}^{2}\right)}}$$

Where the K is the trend of phenology or climate change; N is the number of years in the study period; G_i_ is the phenology in the year i, and i is the annual variable. When K< 0 means phenology is delayed; when K > 0 means is advanced. r_xy[z]_ is the partial correlation coefficient between phenological indicators x and climate factors y when climate metric z is controlled; r_xy_, r_xz_, and r_zy_ represent linear correlation coefficients.

## 3 Results

### 3.1 Characterization and accuracy assessment of fitted NDVI curves

**Figure 3:**
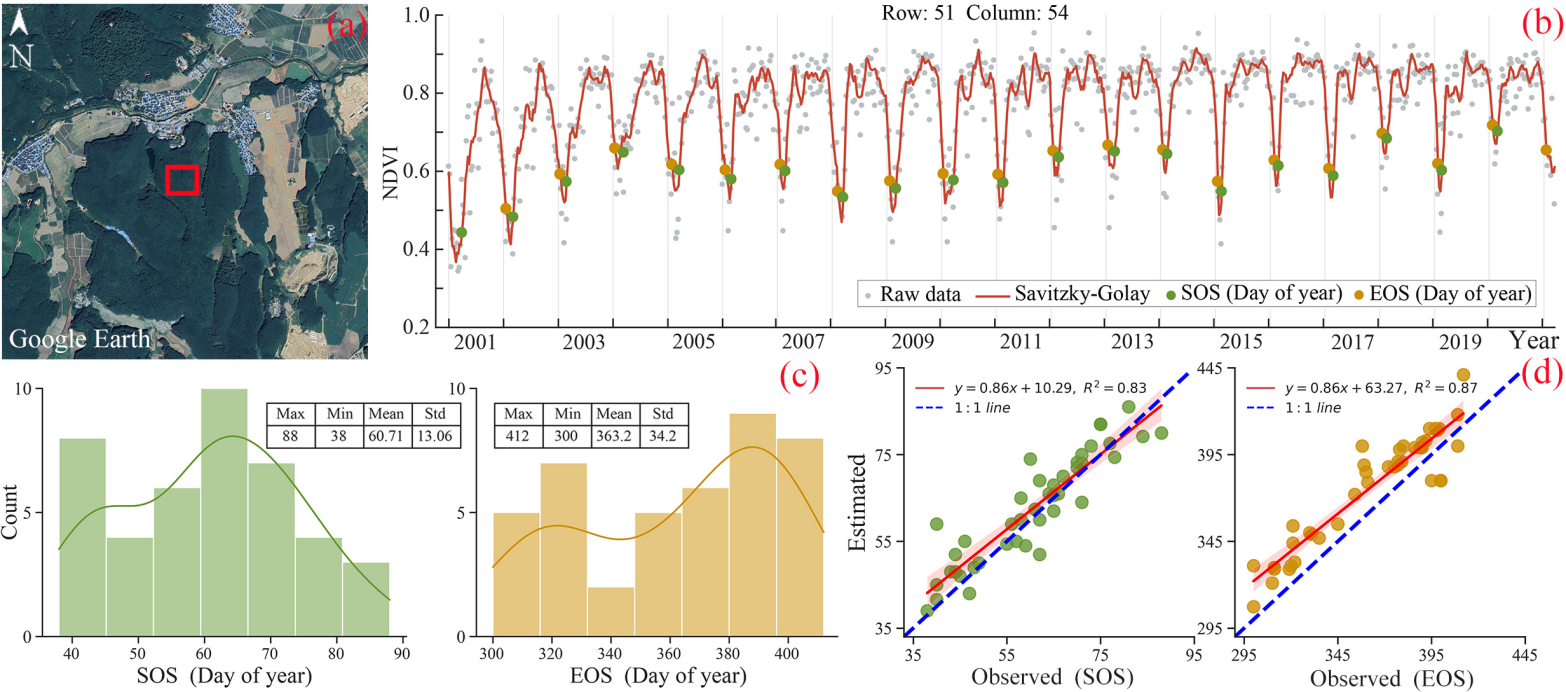
Example of rubber plantations phenological NDVI time-series using LS2 and MODIS imagery (21°31N, 100°39E): **(A)** Google Earth satellite imagery acquired on November 11, 2019, **(B)** NDVI time series, **(C)** Histogram of ground observation data and **(D)** Scatter plot and linear fit of SOS and EOS estimated by remote sensing and field observation.

Using Landsat and Sentinel-2 (LS2) imagery, along with MODIS imagery composite data, we successfully generated high-quality NDVI time series at 8-day intervals with a spatial resolution of 30 meters. To effectively capture the inter-annual variability of rubber phenology and eliminate noise, we chose the S-G filter and applied it to NDVI time series smoothing (**Figures 3A, B**). The distribution of SOS (DOY60 ± 13) showed a marginal left skewness, while the distribution of EOS (360 ± 34) exhibited a right skewness (**Figure 3C**). Validation was carried out by comparing ground-based observations of phenology with remote sensing monitoring of phenological indicators. The obtained R^2^ values for SOS and EOS were 0.83 and 0.87, respectively (**Figure 3D**). Notably, the majority of data points clustered closely to the 1:1 line, indicating a robust linear relationship between the observed and estimated values. To ensure precise and consistent results, the window size, envelope iteration, adaptive strength, and seasonal amplitude thresholds (SOS and EOS) were determined as 5, 2, 3, 15%, and 20%, respectively. These optimized parameters collectively contributed to the extraction of the SOS and EOS, enabling a comprehensive analysis of phenology in rubber plantations.

### 3.2 Spatial-temporal characteristic of rubber phenology

**Figure 4:**
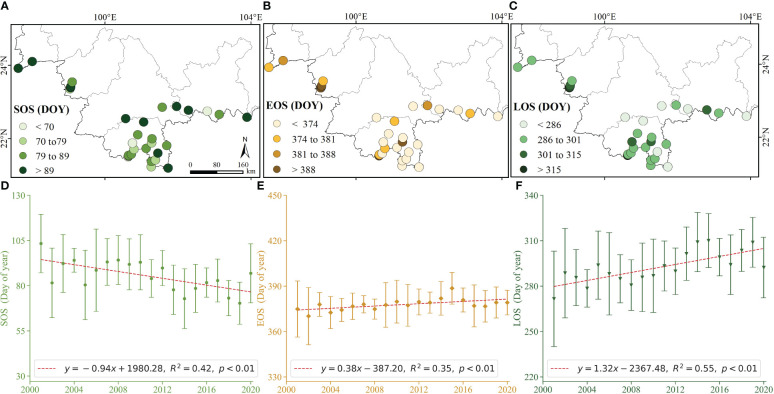
Spatial-temporal variation of phenology indicators: 20-year mean of **(A)** SOS, **(B)** EOS, and **(C)** LOS, and temporal characteristics of **(D)** SOS, **(E)** EOS, and **(F)** LOS, respectively.

**Figures 4A–C** illustrates the spatial distribution of average phenology indicators in rubber plantations spanning from 2001 to 2020. The SOS (DOY 70-89) indicates a concentration in the mid-March. Conversely, EOS (DOY 374-388, DOY values greater than 365 indicate days in the following year) indicates a concentration in the early January. The LOS in rubber plantations was approximately 10 months. Furthermore, spatial distribution of rubber phenology indicators revealed distinct regional variations.

**Figures 4D–F** presents the inter-annual variation in rubber phenology. Over the past 20 years, the SOS demonstrated advancement from DOY 103 ± 16 days in 2001 to DOY 86 ± 16 days in 2020, exhibiting a rate of 9.4 days per decade (R^2^ = 0.42, *p*< 0.01). Conversely, the EOS demonstrated a delay from DOY 374 ± 18 days in 2001 to DOY 379 ± 8 days in 2001, showing a rate of 3.8 days per decade (R^2^ = 0.35, *p*< 0.01). The LOS also exhibited a significant extension from DOY 271 ± 31 days in 2001 to DOY 292 ± 20 days in 2020, reflecting of extended at 13.2 days per decade (R^2^ = 0.55, *p*< 0.01). These trends illustrate the dynamic changes in rubber phenology that have occurred over the past two decades, with both the SOS and EOS being substantially affected.

### 3.3 Relationship between rubber phenology and climate factors

#### 3.3.1 Annual temperature and precipitation

**Figure 5:**
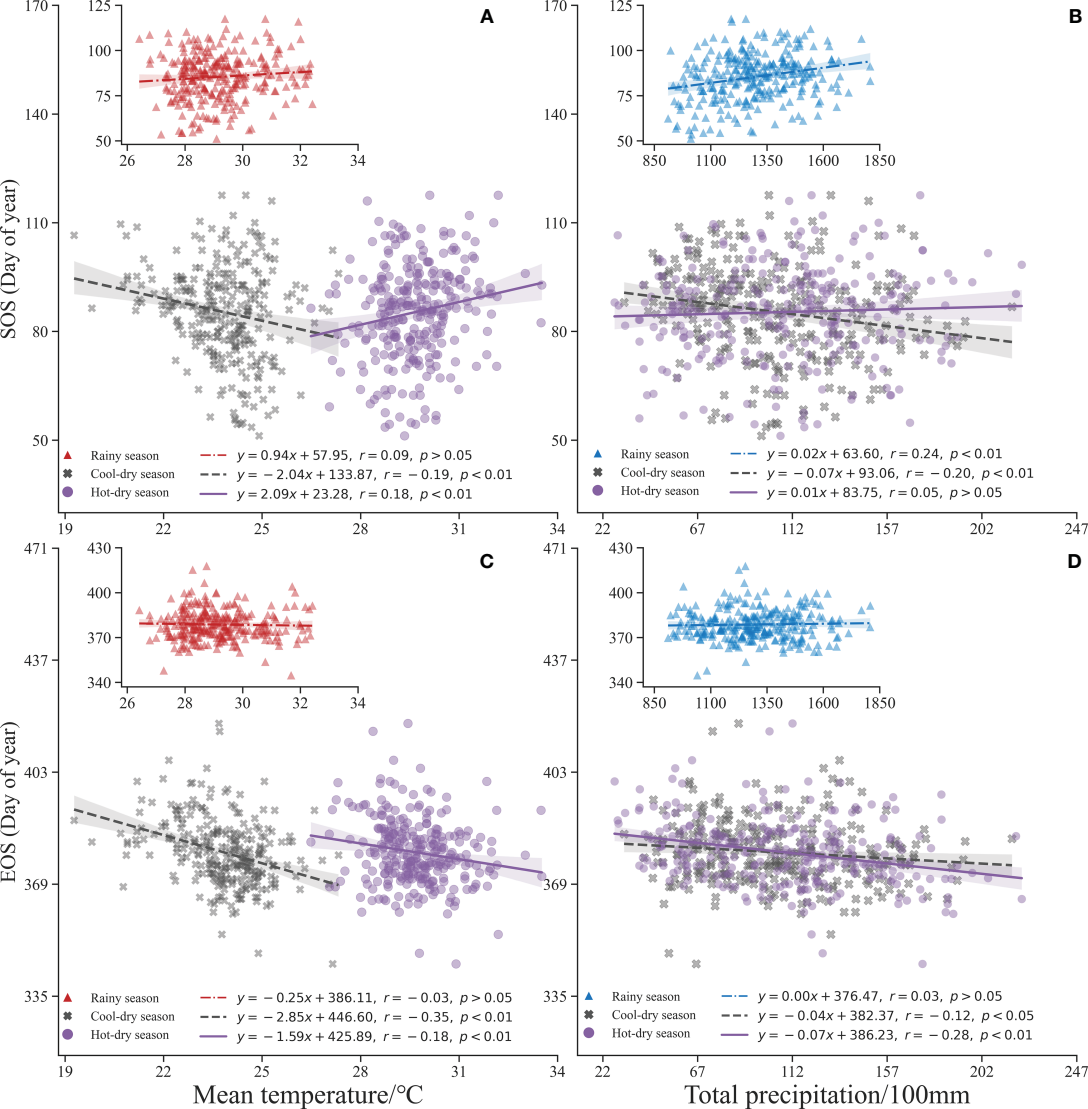
Phenology of rubber plantations versus annual mean temperature and total precipitation: **(A, B)** SOS versus annual mean temperature and total precipitation, and **(C, D)** EOS versus annual mean temperature and total precipitation, respectively.

Temperature during the cool-dry season and precipitation during the rainy season are the primary determinants of rubber SOS (**Figures 5A, B**). Specifically, the SOS advanced by 2.0 days for every 1°C increase in the cool-dry season (r =−0.19, *p<* 0.01). In contrast, the SOS delayed by 2.0 days for every 1°C increase in the hot-dry season (r = 0.18, *p<* 0.01) (**Figure 5A**). Notably, heightened precipitation levels during the rainy season led to a delay in SOS, with a 100 mm increase in rainy season precipitation resulting in a 2.0 days delay (r = 0.24, *p*< 0.01). However, this pattern exhibited variability during the cool-dry season, where each 100 mm increase in precipitation brought about an advancement of SOS by 7.00 days (**Figure 5B**).

Similar patterns are observed for the EOS, with the most influential factors being the temperature during the cool-dry season and precipitation during the hot-dry season (**Figures 5C, D**). A noteworthy advancement of EOS is observed with every 1°C increase in the cool-dry season temperature leading to an advancement of 2.8 days (r =−0.35, *p<* 0.01). This same trend prevails in the hot-dry season, where EOS advances by 1.5 days for every 1°C increase (r =−0.18, *p<* 0.01) (**Figure 5C**). Furthermore, we noted a 7.0 days advancement of EOS for every 100 mm increase in hot-dry season precipitation (r =−0.28, *p*< 0.01). An increment of 100 mm in cool-dry season precipitation results in a 4.0 days advancement of EOS (r =−0.12, *p<* 0.05) (**Figure 5D**).

#### 3.3.2 Preseason temperature and precipitation

**Figure 6:**
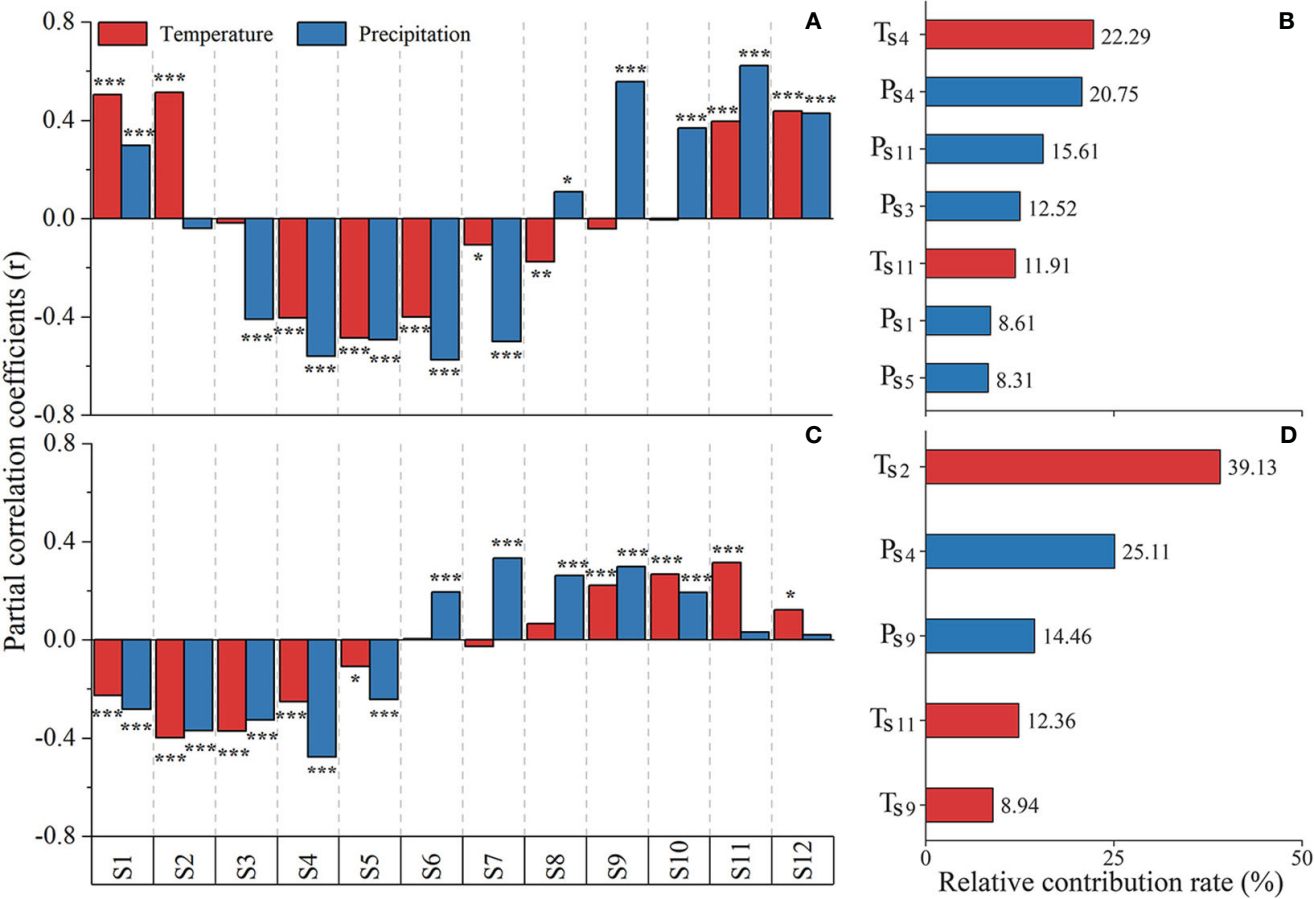
Distribution of partial correlation coefficients and relative contribution rates of dominant factors between phenology and preseason monthly climate: **(A, C)** partial correlation coefficients and **(B, D)** relative contribution rates, respectively. Asterisks ^*^, ^**^, and ^***^ denote *p* values*<* 0.05, 0.01, and 0.001, respectively.

Pearson correlation analysis provided valuable insights into the response of SOS to preseason climate change at a monthly scale. Through the examination of partial correlation coefficients between SOS and climate factors (**Figure 6A**), we observed a positive correlation between SOS and temperature during the second preseason months. However, this correlation progressively shifted to become negative during the subsequent six months. Notably, several partial correlation coefficients exhibited absolute values exceeding 0.5, indicating strong associations. Specifically, as the number of preseason months increased, the impact of higher preseason temperature and precipitation on the delay of SOS gradually shifted towards an advancement.

To identify the primary climatic factors influencing SOS, we conducted multiple stepwise regression analysis, with SOS as the dependent variable and preseason temperature and precipitation as the independent variables, considering their significant correlations. The results of the multiple stepwise regression (**Figure 6B**) revealed that the fourth preseason month temperature made the most relative contribution, accounting for 22.29% of the variation in SOS. The relative contribution of precipitation in the fourth, eleventh and third preseason months was 20.29%, 15.61% and 12.52%, respectively. Consequently, it can be inferred that the temperature in fourth preseason month exerts the most substantial influence on the variation in SOS, followed by precipitation in fourth and eleventh preseason months. The fitted equation for SOS according to equation (5).


(5) $$\begin{array}{c} \text{SOS}=99.568-2.299T_{\text{S}4}-0.122P_{\text{S}4}+0.045P_{\text{S}11}-0.166P_{\text{S}3}+1.386T_{\text{S}11}+0.113P_{\text{S}1}-0.028P_{\text{S}5}\\ R^{2}=0.718,p<0.01 \end{array}$$

Where *T*s4 and *T*s11 denote the temperature in the fourth and eleventh preseason months, and with relative contributions of 22.29%, and 11.91%, respectively; and *P*s4, *P*s11, *P*s3, *P*s1 and *P*s5 denote the precipitation in the fourth, eleventh, third, first, and fifth preseason months, respectively; with relative contributions of 20.75%, 15.61%, 12.52%, 8.61%, and 8.31%, respectively. The equation takes into account the significant influence of these climatic factors on SOS.

There was a pattern between the EOS and preseason temperature and precipitation (**Figure 6C**). Initially, EOS was negatively correlated with temperature and precipitation for approximately six months, with the effect of temperature being more pronounced than the effect of precipitation. Subsequently, it shifted to a positive correlation, and as the increasing of the number of preseason months, the effect of precipitation became more significant.

To determine the dominant climatic factors influencing EOS, we conducted multiple stepwise regression analysis, with EOS as the dependent variable and preseason temperature and precipitation as the independent variables, considering their significant correlations. The results of the multiple stepwise regression (**Figure 6D**) revealed that the second temperature, fourth precipitation, and ninth precipitation preseason month made the most significant contribution, with respective relative contributions of 39.13%, 25.11%, and 14.46% of the variation in EOS. Additionally, second preseason month in temperature was the primary cause of EOS variation, followed by the fourth and ninth preseason month in precipitation. The fitted equation for EOS according to equation (6).


(6) $$\begin{array}{c} \text{EOS}=413.184-2.882T_{S2}-0.039P_{S4}+0.046P_{S9}+0.686T_{S11}+0.728T_{S9}\\ R^{2}=0.419,p<0.01 \end{array}$$

Where *T*s2, *T*s11, and *T*s9 denote the temperature in the second, eleventh, and ninth preseason months, and with relative contributions of 39.13%, 12.36%, and 8.94%, respectively; and *P*s4 and *P*s8 denote the precipitation in the fourth and eighth preseason months, respectively; with relative contributions of 25.11% and 14.46%, respectively.

## 4 Discussion

### 4.1 Phenology monitoring using LS2 and MODIS images

The selection of satellite imagery with appropriate spatial resolution is critical for phenological monitoring (Cheng et al., 2020; Zhang et al., 2022). The use of coarse-resolution imagery, despite its enhanced temporal resolution, introduces difficulties in monitoring landscapes characterized by high fragmentation due to the problem of mixed pixel interpretation. Moreover, frequent occurrences of cloud cover and precipitation in tropical regions can impede the establishment of consistent time series data derived from a single satellite sensor (Cleland et al., 2007; Azizan et al., 2021a; Chen G. et al., 2023). To mitigate these challenges, our study employs medium-resolution Landsat and Sentinel-2 imagery, thereby augmenting spatial resolution and ensuring continuity in time series data. The decision to utilize 30-m resolution image data, as opposed to the traditional 250-m resolution MODIS data, enhances the precision of our analysis significantly (Cheng et al., 2020; Yang et al., 2023). Although Sentinel-2 has been operational only since 2017, its integration efficaciously complements Landsat data and precludes discontinuities in time series data, thereby presenting invaluable opportunities for the monitoring of rubber phenology (Chastain et al., 2019; Li et al., 2022).

The defoliation to refoliation transition in rubber trees typically spans approximately 6 to 8 weeks. In light of this, the creation of 8-day interval NDVI time-series data was undertaken in this study to provide a more granular perspective on key phenological dynamics (Zhai and Xu, 2022). The S-G filter method from Timesat3.3 software was utilized to eliminate noise (Azizan et al., 2022). Although thresholds of seasonal amplitude differ from previous rubber phenology, studies conducted in Sumatra, Indonesia (20% and 20%) by Azizan et al. (2021a) and Hainan Island, China (30% and 60%) by (Hu et al., 2022), exhibit greater stability in the context of the Yunnan rubber plantation. This stability is crucial because it lends more reliability to the remote sensing monitoring of rubber phenology, offering valuable insights that could drive future research and inform the management of rubber plantation more effectively (Pastick et al., 2020).

### 4.2 Spatial and temporal heterogeneity of rubber phenology

This study demonstrates distinct spatial and temporal regional differences in the phenology of rubber plantations in the context of global climate change (**Figure 4**). In particular, the SOS (DOY 70-89) and EOS (DOY 374-388) fall within the normal range of the dry season. This pattern is consistent with the annual defoliation (EOS) occurring in January and February, followed by refoliation (SOS) in February and March for rubber trees in Xishuangbanna (Priyadarshan, 2017; Zhai et al., 2019; Zhai and Xu, 2022). Additionally, the inter-annual variability of rubber plantations displays diverse trends, with SOS being advanced at a rate of 9.4 days per decade (R^2^ = 0.42, *p*< 0.01), while EOS being delayed at a rate of 3.8 days per decade (R^2^ = 0.35, *p<* 0.01), and LOS being extended at a rate of 13.2 days per decade (R^2^ = 0.55, *p*< 0.01) (**Figures 4D–F**). These findings support the notion that global warming has a discernible impact on phenology in the tropical Northern Hemisphere, aligning with trends observed in phenological characteristics on Hainan Island (Chen et al., 2016; Zhai et al., 2019; Li N. et al., 2020; Zhai and Xu, 2022). The comprehensive coverage of the Yunnan rubber plantation area in this study provides more robust data, serving as a basis for further quantitative assessments (Merrick et al., 2019; Azizan et al., 2021b). The spatial-temporal variation in rubber phenology is influenced by numerous factors (Azizan et al., 2022). Beyond climatic factors, topographic characteristics, stand age, clone, planting density, nutrient conditions, and other variables can impact phenology (Carr, 2012; Rivano et al., 2016; Azizan et al., 2021a). For instance, topography, such as uphill and higher altitudes, influences early defoliation and phenological fluctuations (Montag, 2017). Different rubber clone also exhibits varied phenology, as demonstrated by PB 86 in Sri Lanka exhibit earlier SOS and EOS compared to mature rubber (Zapata-Gallego and Álvarez-Láinez, 2019). RRIM 600 and GT 1 defoliated and refoliated one to two weeks earlier than clones Yunyan 277-5, Yunyan 34-4, and PR 107, and these two clones also have shorter wintering periods compared to the other three clones (Liyanage et al., 2019). Planting density and stand age are impact phenology, with rubber mix spacing ranging from 4 to 12 m (Lin et al., 2022). Disease outbreaks, like springtime powdery mildew, can lead to substantial leaf shedding and a delay in SOS (Guyot et al., 2001; Kou et al., 2015; Zhai et al., 2019; Zhai et al., 2023). Furthermore, soil nutrient availability plays a role, the soil stores nutrients from decomposing leaves for use by the trees during the refoliation periods (Li Y. et al., 2016). In conclusion, further research is needed to comprehensively understand the multifaceted factors influencing the spatial and temporal aspects of rubber phenology (Liu X. et al., 2013).

### 4.3 Response of rubber phenology to temperature and precipitation

#### 4.3.1 Annual temperature and precipitation

**Figure 7:**
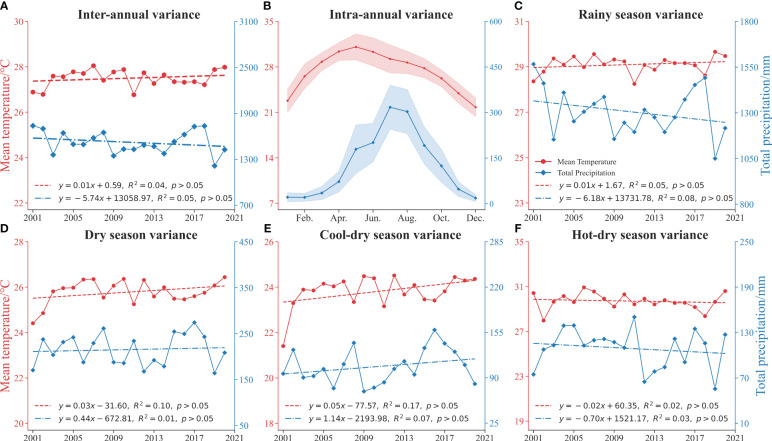
Annual and season variation of mean temperature and total precipitation: **(A)** Inter-annual variance, **(B)** Intra-annual variance, **(C)** Rainy season variance, **(D)** Dry season variance, **(E)** Cool-dry season variance, **(F)** Hot-dry season variance.

Principal factors influencing the SOS and EOS are temperature during the dry season and precipitation during the rainy season. The SOS advanced 2.04 days (r =−0.19, *p*< 0.01) and the EOS advanced 2.85 days (r =−0.35, *p*< 0.01) for every 1°C rise in the cool-dry season. The SOS was delayed by 2.00 days for every 100 mm increase in precipitation during the rainy season (r = 0.24, *p*< 0.01), whereas the EOS was advanced by 7.00 days for every 100 mm increase in precipitation during the hot-dry season (r =−0.28, *p*< 0.01) (**Figure 5**). During the dry season (November to April), these trees experience a rapid SOS and EOS (**Figures 7A, B, D**) (Yeang, 2007; Liu et al., 2016; Liyanage et al., 2019). The rainy season (May to October) in the study area exhibits a fluctuating trend of decreasing precipitation, with more than 80% of precipitation concentrated in the rainy season, precipitation decreased during the rainy season causing the SOS to advance (**Figure 7C**) (Zhai and Xu, 2022). Temperature and precipitation trends vary between the cool-dry season (November to February) and the hot-dry season (March to April) (**Figures 7E, F**). With an average temperature of about 21°C during the hot-dry season and a decrease in precipitation, EOS was delayed (**Figure 7F**) (Zhai et al., 2019). These findings illustrate the impact of global warming on climate dynamics (Li Y. et al., 2016; Zhai et al., 2021).

As seasonal plants, rubber trees are extremely sensitive to cool-dry temperature variations, which have a direct effect on their growth rate (**Figures 5A, C**) (Zhai et al., 2020). The preponderance of biochemical processes in ectotherms, such as reaction kinetics, enzyme inactivation, and hormonal regulation, are affected by changes in temperature (Lee et al., 2020). Temperature increases during the cool-dry season not only stimulate the growth and metabolic processes of rubber, but also increase the rate of water evaporation (**Figure 7E**) (Priyadarshan, 2017; Li et al., 2018). This further advances the SOS, which is consistent with the fact that drought stress triggered an earlier EOS (Li Y. et al., 2016). Conversely, decreasing of precipitation during the monsoon season can have a delaying effect on rubber phenology (**Figure 7C**) (Liyanage et al., 2019; Zhai et al., 2019). By alleviating soil moisture stress and increasing leaf photosynthesis rates, and increased rainfall during the cool-dry season effectively mitigates the effects of drought stress on phenology (Zhai and Xu, 2022). Another intriguing aspect of rubber phenology lies in the legacy effect of EOS on SOS (Liyanage et al., 2016; Gutiérrez-Vanegas et al., 2020). Extreme precipitation events during the rainy season should be viewed with caution, as they may have negative effects on rubber (Zhai et al., 2019).

#### 4.3.2 Preseason temperature and precipitation

The selection of an appropriate preseason month is crucial for understanding how climatic factors influence plant phenology (Güsewell et al., 2017; Azizan et al., 2022). As the number of preseason months increased, the influence of preseason temperature and precipitation on the delay of SOS gradually shifted towards advancement (**Figure 6A**). During the wintering period (December-January), powdery mildew is prone to occur in high-temperature and humid climates. When the disease is severe, it can cause rubber leaves to fall, thereby delaying SOS (Zhai et al., 2020). These findings align with previous research by Azizan et al. (2022), who highlighted the significance of the 90-day preseason climate in determining whether rubber tree phenology advanced or delayed. Notably, the variation in SOS is primarily driven by changes in temperature during the fourth preseason month, and precipitation during the fourth and eleventh preseason month. The combination of these factors alleviates soil moisture stress and leads to an advancement of SOS (Rao et al., 1998; Chen et al., 2018; Azizan et al., 2021a). Interestingly, our results differ from the SOS advances in Xishuangbanna, China, during December due to lower temperatures (Zhai et al., 2021). We found that SOS is subject to the complex interplay of various environmental factors, and the impact of temperature on SOS may vary in different regions (Chen et al., 2018). Therefore, further investigations and field trials are warranted to unravel regional differences and provide a more comprehensive understanding of the factors influencing SOS in rubber plantations in Yunnan province (Samplonius et al., 2018; Chen et al., 2020; Wu et al., 2021; Lin et al., 2022).

As the number of preseason months increased, the delayed effects of preseason temperature reduction and precipitation reduction on EOS gradually shift to advance (**Figure 6C**). The analysis of preseason months revealed that EOS was notably influenced in temperature variations during second, fourth, and ninth among the preseason months, correspond to the dry seasons, exhibiting a negative correlation with EOS for the second and fourth preseason month, and a positive correlation with EOS for the ninth preseason month (**Figures 6C, D**). The impact of preseason temperature on EOS is further highlighted by the observed delay in EOS, which could be attributed to either the lower temperatures occurring in October to November (before latex harvesting cessation) or the higher temperatures in December to January (after latex harvesting cessation) (Zhai and Xu, 2022). The dropping temperatures and decreasing precipitation during the hot-dry season may subject rubber trees to drier conditions (**Figure 7F**), the optimal temperature range for rubber tree growth falls between 20°C and 22 °C (Yeang, 2007; Liu et al., 2016; Liyanage et al., 2019). The decrease in precipitation and maintenance of favorable sunlight conditions were advantageous to the photosynthesis of rubber trees and further delayed EOS (Li et al., 2018; Gutiérrez-Vanegas et al., 2020; Li et al., 2021). Cold stress is identified as a primary factor causing defoliation in rubber trees, with severe defoliation occurring within a relatively short time frame in the Xishuangbanna (Liu W. et al., 2013; Lin et al., 2018). Furthermore, the progression of EOS may also be influenced by the reduction in daylight length during the rainy season and the cold dry period (Omokhafe and Alika, 2003; Liyanage et al., 2019). In summary, the impact of preseason temperature on the EOS is evident during specific months, with dry seasons playing a significant role (Zhai and Xu, 2022).

Despite the valuable insights gained from this investigation, there are certain limitations that need to be acknowledged (Lin et al., 2022). Firstly, the study was conducted within a specific geographical sample point and was limited to two decades (Fu et al., 2018; Azizan et al., 2021a). To gain a more comprehensive understanding of rubber tree phenology, future research could expand the scope by incorporating data from multiple locations and exploring additional remote sensing data and advanced processing methods (Yeang, 2007; Zhang et al., 2022). Secondly, while our study focused on the influence of climatic factors on rubber tree phenology, it is crucial to recognize that other factors, such as human activities, species-specific characteristics, and policy regulations, may also exert a combined effect on phenology (Priyadarshan, 2017; Lin et al., 2018). The interactions and cumulative impact of these various factors deserve further investigation to better grasp the intricate dynamics that govern rubber tree phenological patterns (Kou et al., 2015; Ali et al., 2020; Hazir et al., 2020).

## 5 Conclusion

Understanding the phenology of rubber plantations is pivotal for both production management and for assessing the implications of climate change within tropical regions. In this study, we adopted an approach leveraging dense satellite imagery time series to monitor the spatiotemporal dynamics of rubber phenology, facilitating a quantified assessment of its climate response. We developed an algorithm for creating phenology monitoring datasets (30-m resolution and 8-day intervals) by combining Landsat, Sentinel-2, and MODIS time-series imagery and used S-G filter to reduce noise. The results indicated that both the SOS (DOY70-89) and EOS (DOY374-388) of rubber plantation fall within the normal range of the dry season. Over the past two decades, there has been a significant advancement of the SOS by 9.4 days per decade (R^2^ = 0.42, *p*< 0.01), a delay of EOS by 3.8 days per decade (R^2^ = 0.35, *p*< 0.01), and an extension of the LOS by 13.2 days per decade (R^2^ = 0.45, *p*< 0.01).

The principal determinants of rubber SOS are temperature during the cool-dry season and precipitation during the rainy season. Specifically, the SOS advanced 2.0 days for every 1°C increase in the cool-dry season (r =−0.19, *p<* 0.01), whereas a 100 mm increase in rainy season precipitation caused a 2.0 days delay (r = 0.24, *p*< 0.01). Similar patterns are observed for the EOS, with temperature during the cool-dry season and precipitation during the hot-dry season being the most influential factors. With every 1°C increase in cool-dry season temperature, the EOS advances by 2.8 days (r =−0.35, *p*< 0.01), and for every 100 mm increase in hot-dry season precipitation, the EOS advances by 7.0 days (r =−0.28, *p*< 0.01). Our study detected a sensitive phenological response to preseason climate variations in rubber plantations, suggesting a legacy effect. As the number of preseason months increased, the influence of heightened preseason temperature and precipitation shifted from an advancing to a delaying effect on SOS and EOS. While climatic variables play an integral role, it is important to note that phenology is also influenced by non-climatic factors, such as topography, stand age, planting density, and nutrient conditions.

## Data availability statement

The original contributions presented in the study are included in the article/**Supplementary Material**. Further inquiries can be directed to the corresponding authors. Correspondence and requests for materials should be addressed to BC and HL.

## Author contributions

HL: Conceptualization, Data curation, Investigation, Methodology, Supervision, Validation, Visualization, Writing – original draft, Software. BC: Conceptualization, Funding acquisition, Investigation, Methodology, Resources, Validation, Visualization, Writing – review & editing. XY: Investigation, Methodology, Software, Validation, Visualization, Writing – review & editing. GW: Formal analysis, Investigation, Validation, Writing – review & editing. XW: Data curation, Methodology, Software, Validation, Visualization, Writing – review & editing. TY: Data curation, Formal analysis, Funding acquisition, Project administration, Software, Visualization, Writing – review & editing. GL: Data curation, Formal analysis, Software, Visualization, Writing – review & editing. ZW: Formal analysis, Investigation, Methodology, Project administration, Software, Supervision, Validation, Visualization, Writing – review & editing. CY: Conceptualization, Formal analysis, Investigation, Methodology, Visualization, Writing – review & editing. WK: Conceptualization, Data curation, Formal analysis, Funding acquisition, Project administration, Resources, Supervision, Writing – review & editing.
