# Supplementary material for: Dry season temperature and rainy season precipitation significantly affect the spatio-temporal pattern of rubber plantation phenology in Yunnan province
Source: Front Plant Sci. 2023 Dec 14;14:1283315. doi: 10.3389/fpls.2023.1283315 (PMC10752945; doi:10.3389/fpls.2023.1283315)
Supplement: Supplementary file 1 [file DataSheet_1.docx]

**Supplementary information for**

**Dry season temperature and rainy season precipitation significantly affect the spatio-temporal pattern of rubber plantation phenology in Yunnan Province**

Hongyan Lai^1,2^, Bangqian Chen^2*^, Xiong Yin^1,2^, Guizhen Wang^2^, Xincheng Wang^2,3^, Ting Yun^3^, Guoyu Lan^2^, Zhixiang Wu^2^, Chuan Yang^2^, Weili Kou^1*^^[[1]](#footnote-1)^

1. College of Forestry, Southwest Forestry University, Kunming 650233, China;
2. Hainan Danzhou Agro-ecosystem National Observation and Research Station; State Key Laboratory Incubation Base for Cultivation & Physiology of Tropical Crops; Rubber Research Institute (RRI), Chinese Academy of Tropical Agricultural Sciences (CATAS) Haikou 571101, China.
3. Co-Innovation Center for Sustainable Forestry in Southern China, Nanjing Forestry University, Nanjing 210037, China.

# **Figures**


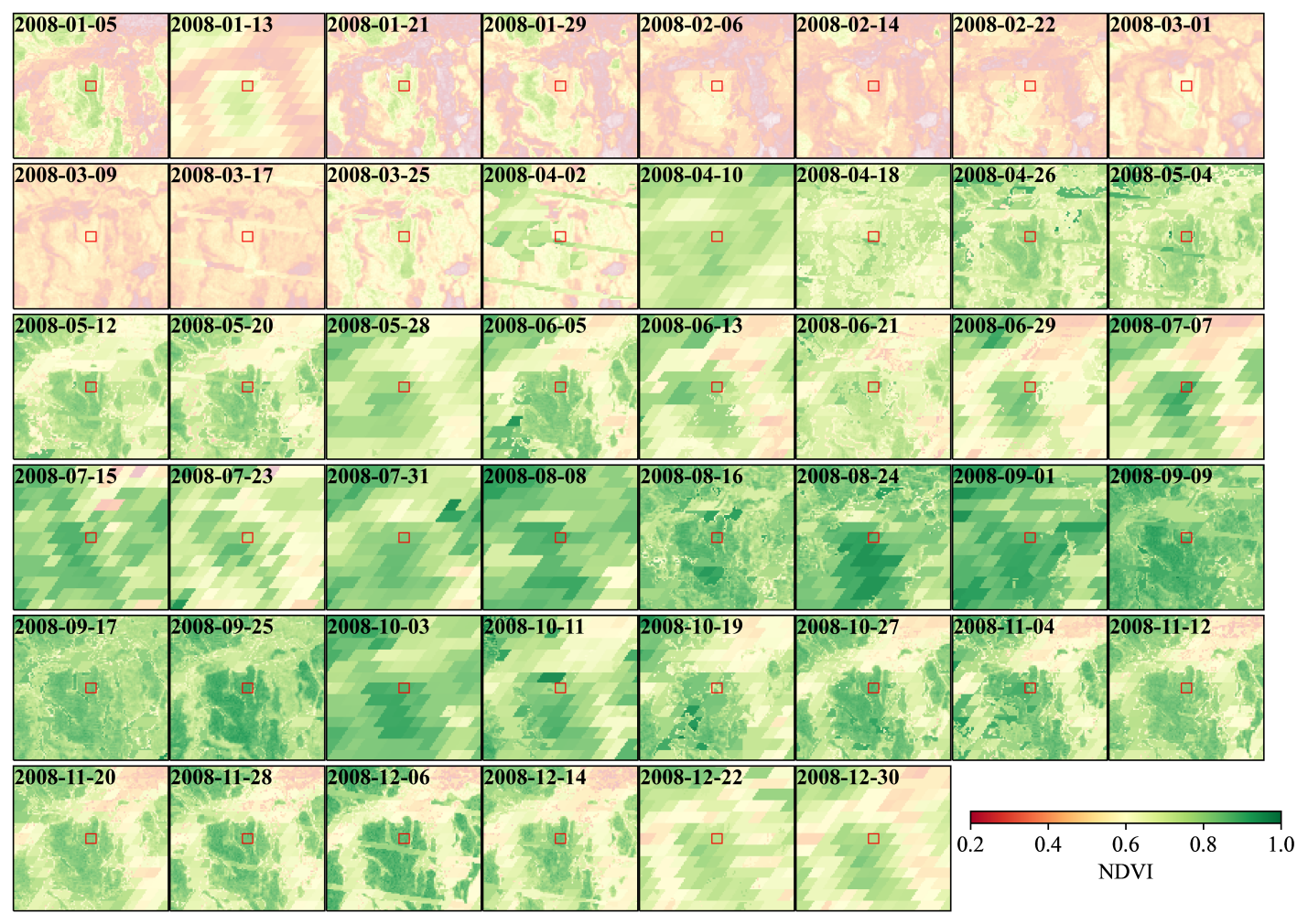


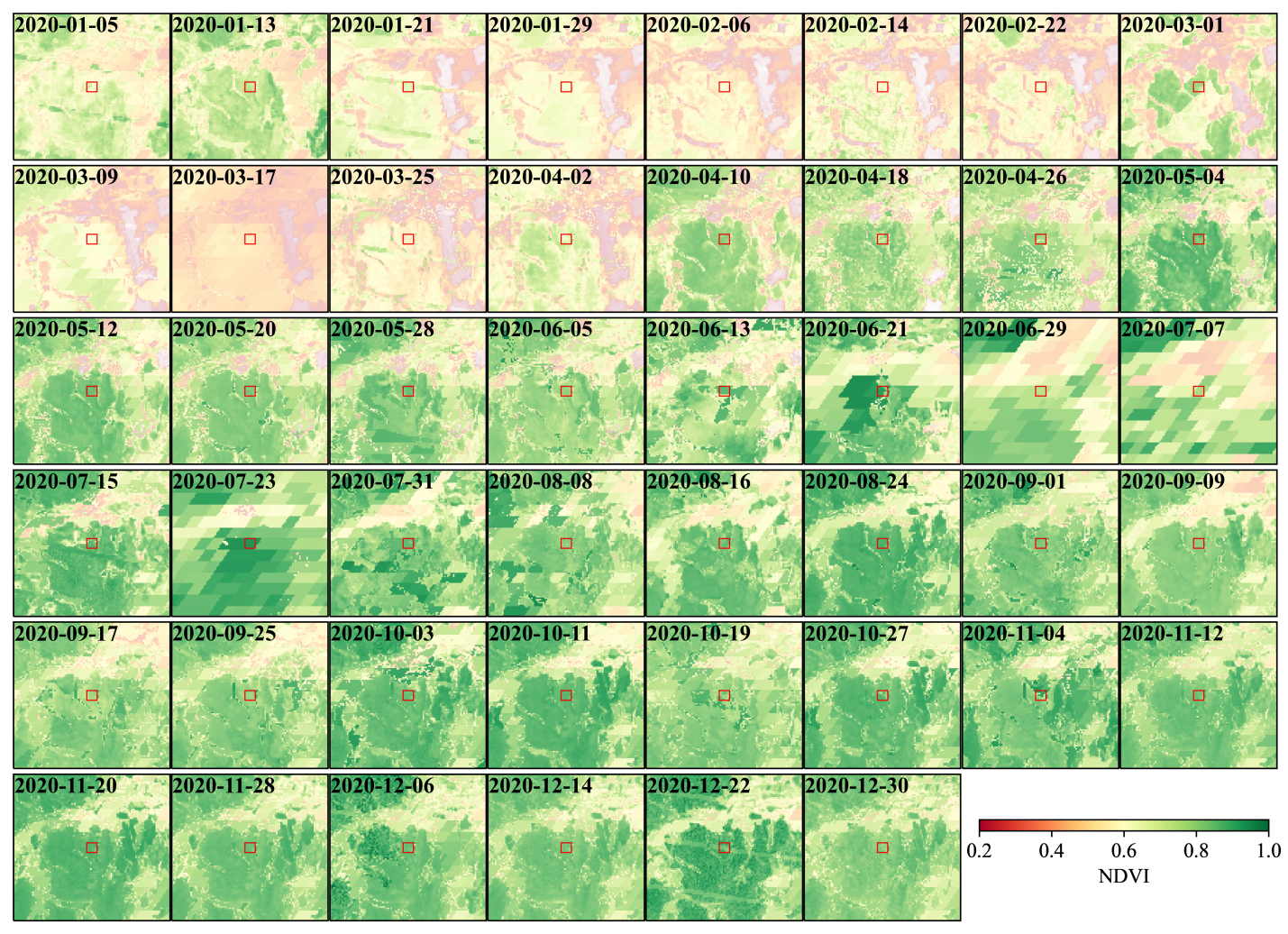


**Fig. S1.** An example of NDVI time-series in 2008 and 2020 composited from LS2 and MODIS imagery (21°48 N, 100°46E) in a sub-optimal environment in Yunnan, Southwest China.

1. ^*^Corresponding authors: chbq40@163.com (Chen, B.), kwl_eric@163.com (Kou, W.) [↑](#footnote-ref-1)
